# Mechanisms underlying epigenetic and transcriptional heterogeneity in Chinese hamster ovary (CHO) cell lines

**DOI:** 10.1186/s12896-016-0238-0

**Published:** 2016-01-22

**Authors:** Nathalie Veith, Holger Ziehr, Roderick A. F. MacLeod, Stella Marie Reamon-Buettner

**Affiliations:** Pharmaceutical Biotechnology, Fraunhofer Institute for Toxicology and Experimental Medicine, Inhoffenstrasse 7, 38124 Braunschweig, Germany; Leibniz Institute DSMZ-German Collection of Microorganisms and Cell Cultures, Inhoffenstrasse 7B, 38124 Braunschweig, Germany; Preclinical Pharmacology and In Vitro Toxicology, Fraunhofer Institute for Toxicology and Experimental Medicine, Nikolai-Fuchs Strasse 1, 30625 Hannover, Germany

**Keywords:** CHO cells, Recombinant protein production, Chromatin, Epigenetic silencing, DNA methylation, Histone modifications

## Abstract

**Background:**

Recombinant cell lines developed for therapeutic antibody production often suffer instability or lose recombinant protein expression during long-term culture. Heterogeneous gene expression among cell line subclones may result from epigenetic modifications of DNA or histones, the protein component of chromatin. We thus investigated in such cell lines, DNA methylation and the chromatin environment along the human eukaryotic translation elongation factor 1 alpha 1 (*EEF1A1*) promoter in an antibody protein-expression vector which was integrated into the Chinese hamster ovary (CHO) cell line genome.

**Results:**

We analyzed four PT1-CHO cell lines which exhibited losses of protein expression at advanced passage number (>P35) growing in adherent conditions and in culture medium with 10 % FCS. These cell lines exhibited different integration sites and transgene copy numbers as determined by fluorescence in situ hybridization (FISH) and quantitative PCR (qPCR), respectively. By qRT-PCR, we analyzed the recombinant mRNA expression and correlated it with DNA methylation and with results from various approaches interrogating the chromatin landscape along the *EEF1A1* promoter region. Each PT1-CHO cell line displayed specific epigenetic signatures or chromatin marks correlating with recombinant mRNA expression. The cell line with the lowest recombinant mRNA expression (PT1-1) was characterized by the highest nucleosome occupancy and displayed the lowest enrichment for histone marks associated with active transcription. In contrast, the cell line with the highest recombinant mRNA expression (PT1-55) exhibited the highest numbers of formaldehyde-assisted isolation of regulatory elements (FAIRE)-enriched regions, and was marked by enrichment for histone modifications H3K9ac and H3K9me3. Another cell line with the second highest recombinant mRNA transcription and the most stable protein expression (PT1-7) had the highest enrichments of the histone variants H3.3 and H2A.Z, and the histone modification H3K9ac. A further cell line (PT1-30) scored the highest enrichments for the bivalent marks H3K4me3 and H3K27me3. Finally, DNA methylation made a contribution, but only in the culture medium with reduced FCS or in a different expression vector.

**Conclusions:**

Our results suggest that the chromatin state along the *EEF1A1* promoter region can help predict recombinant mRNA expression, and thus may assist in selecting desirable clones during cell line development for protein production.

**Electronic supplementary material:**

The online version of this article (doi:10.1186/s12896-016-0238-0) contains supplementary material, which is available to authorized users.

## Background

Cell lines combining high-production and stability are important for recombinant protein production, notably of therapeutic antibodies. These antibodies are chiefly produced in Chinese hamster ovary (CHO) cells which combine several advantageous qualities, notably that these antibodies are compatible with humans and bioactive therein [1]. However, the development of high-producing recombinant cell lines in CHO cells is laborious as well as cost-intensive. From the delivery of the recombinant DNA into the host cell nucleus for chromosomal integration, to several rounds of screening and selection of high-producing clones, and until commercial manufacturing can take many months. More importantly, such high-producing cell line subclones often manifest heterogeneous expression patterns or lose expression of the recombinant protein during a long-term culture. Thus, loss of productivity is a chronic problem which reflects the operation of multiple causes [2–4]. Nevertheless, the exact mechanisms underlying subclonal variations and genomic instability are still not well understood. Processes known to contribute to overall recombinant protein production stability include transcription, translation, protein folding, and protein secretion. Hence, a wide range of strategies encompassing practically all aspects of cell line development and cultivation in recombinant protein production in CHO cells is used to mitigate this problem [5].

Chromatin is a complex nucleoprotein structure in which the DNA is packaged in the cell nucleus. At the chromatin level, different epigenetic events operate that can affect the integration sites of the protein-expression vector into the CHO genome. Thus, epigenetic events may contribute to the transcriptional repression of the transgene [6, 7]. The known epigenetic effectors include DNA methylation, nucleosome positioning, histone variants, histone modifications, and non-coding RNAs. These could function independently or combinatorially to affect recombinant mRNA and ipso facto protein expression [8]. Thus for example, a modification by DNA methylation through the addition of a methyl group to the C5 carbon residue of cytosines in the C-G dinucleotide (known as CpGs) in the promoter region driving the transgene can effect silencing in several ways. Transcriptional repression by DNA methylation may result through occlusion of transcriptional activator binding to target DNA or recruitment of methyl-CpG-binding domain (MBD) proteins [9]. These MBD proteins recruit modifying and chromatin-remodelling complexes to methylated sites. DNA methylation may also contribute to inhibition of gene expression by promoting a more compact and rigid nucleosome structure [10]. Moreover, DNA methylation in other regions such as gene bodies may also play a role, but so far the precise mechanisms in modulating transcription have yet to be defined [11].

A transgene in the CHO genome can also be influenced by the positioning of the nucleosome at the integration site. The nucleosome is the fundamental repeating chromatin subunit comprised of eight histones encompassed by circa 147 bp of DNA in 1.65 superhelical turns. The histone octamer itself comprises two copies each of histones H2A, H2B, H3, and H4. Nucleosome positioning is key to higher-order chromatin folding and transcriptional regulation [12–14]. Nucleosomes modulate the accessibility of DNA to regulatory proteins and transcriptional machinery to control gene activation or repression. Several factors can affect nucleosome positioning. These include DNA sequence preferences, DNA methylation status, histone variants, and histone post-translational modifications [12]. Replacement of nucleosomal histones with histone variants can influence nucleosome positioning, and thus gene activity [15]. Moreover, a number of post-translational modifications (PTMs) of amino-acid residues in the N-terminals of histones (canonical as well as variants) can affect the epigenetic regulation of chromatin structure and gene function [16]. These PTMs such as acetylation, methylation, ubiquitination and phosphorylation, can determine chromatin state by directly influencing structure or serve as signals to readers of histone modifications [17].

A number of studies have shown that aberrant DNA methylation [4, 18, 19] and histone H3 hypoacetylation [20] exacerbate productivity losses in monoclonal antibody-producing CHO cell lines. Such concerns prompted our current aim to measure the impact of epigenetic silencing mechanisms on promoting clonal heterogeneity during cell line development and protein production after long-term culture. We investigated DNA methylation and used a variety of approaches to interrogate the chromatin environment around the human eukaryotic translation elongation factor 1 alpha 1 (*EEF1A1*) promoter sequence in four recombinant CHO cell lines which exhibited loss of productivity during long-term culture. We found each cell line exhibited chromatin marks highly associated with recombinant mRNA expression. Understanding chromatin environment in recombinant CHO cell lines should help facilitate selection of stable and productive cell lines for recombinant protein production.

## Results

### Characteristic features of the PT1-CHO cell lines

**Fig. 1:**
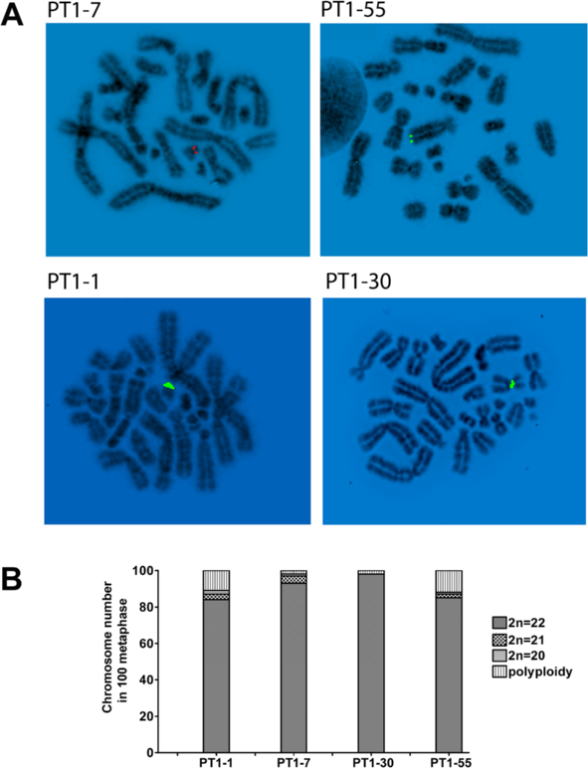
Cytogenetic characterization of PT1-CHO cell lines. **a** FISH analysis showing the different integration sites of the PT1 expression vector in the CHO-K1 genome. A centromeric integration site can be seen for PT1-30, and telomeric for PT1-55. FISH was performed on early passage (P10) recombinant PT1-CHO cell lines. Preparations were counterstained with DAPI (4′,6-Diamidine-2′-phenylindole dihydrochloride). Reverse DAPI conversions were performed to render latent G-banding images visible. Vector DNAs depicted were labelled by nick translation with red emission Dy-590-dUTP (PT1-7), or green emission Dy-495 (PT1-1/30/55) **b** Chromosome number (i.e. 2*n* = 22) ranged from 84 to 98 % as determined in 100 metaphases at passage P55.

In this study, we investigated four PT1-CHO cell lines which exhibited attenuated recombinant protein production after a long-term culture (at passage > P35). These cell lines were developed for antibody production by transfecting CHO-K1 cells with a plasmid-expression-vector construct (designated PT1) carrying cDNA encoding heavy and light chains of a murine IgG2a antibody. As shown by fluorescence in situ hybridization (FISH) analysis, integration sites of the transgene may involve different chromosome regions and chromosomes (Fig. 1a). For instance, FISH performed on short-term cultures (P10) showed a centromeric integration site for PT1-30, while a telomeric one for PT1-55. FISH signals for PT1-1 and PT1-7 were observed in one of the smaller chromosomes of CHO-K1. In particular, PT1-7 was integrated in Z12 at chromosome band p11.

**Fig. 2:**
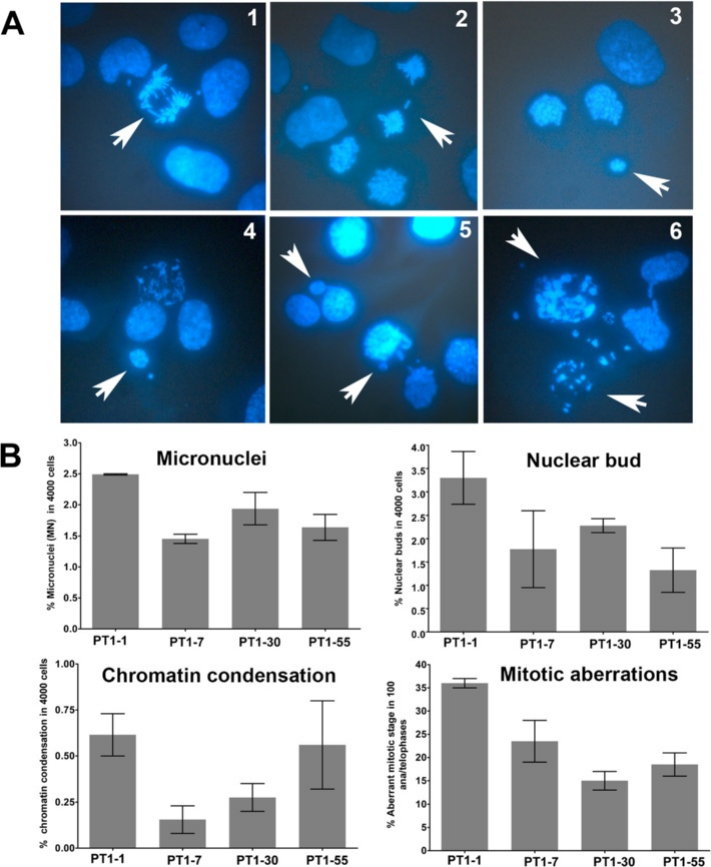
Determination of genomic instability in the PT1-CHO cell lines. **a** Examples of chromosomal and chromatin abnormalities observed after DAPI-staining. Shown are those seen from PT1-30; indicated by arrow(s): (**1**) chromatin bridges at anaphase; (**2**) a lagging chromosome at late anaphase; (**3**) a micronucleus at telophase; (**4**) a micronucleus beside a smaller one; (**5**) nuclear buds; (**6**) chromatin condensation/fragmentation. **b** Frequencies of micronuclei, nuclear buds, chromatin condensation and ana/telophase abnormalities, showing higher frequencies for PT1-1 than the other three cell lines. One-way ANOVA tests for micronucleus formation (*P = 0.0457), and ana/telophase abnormalities (*P = 0.0221) were found significant. Cells were grown in 1-well chamber slides. The frequencies for micronucleus, nuclear bud, and chromatin condensation/fragmentation were determined from *n* = 4000 cells. The frequency for abnormal mitotic stages were determined in *n* = 100 ana/telophases. Data represent means and standard error of the means (SEM) of measurements of two passages (P55 and P72).

The modal chromosome number (2*n* = 22) determined at high passage (P55) in these PT1-CHO cell lines was found in 84–98 % in 100 analyzed metaphases (see Fig. 1b). We also assessed additional indicators of genomic instability at two time points (P55 and P72) such as chromatin abnormalities (premature condensation, fragmentation); micronuclei (MN) and nuclear bud formation (NBUDs); as well as chromosome lagging and chromatin bridges at anaphase and telophase (Fig. 2a). Mean MN frequency ranged from 1.46–2.49 %; NBUDs 1.33–3.30 %; and premature chromatin condensation 0.15–0.62 % as determined in 4000 cells per analysis. Mean frequencies of mitotic aberrations ranged from 15 to 36 % in 100 ana/telophases per analysis. Overall, the PT1-1 cell line exhibited the highest frequencies in the indicators of genomic instability used (Fig. 2b).

**Fig. 3:**
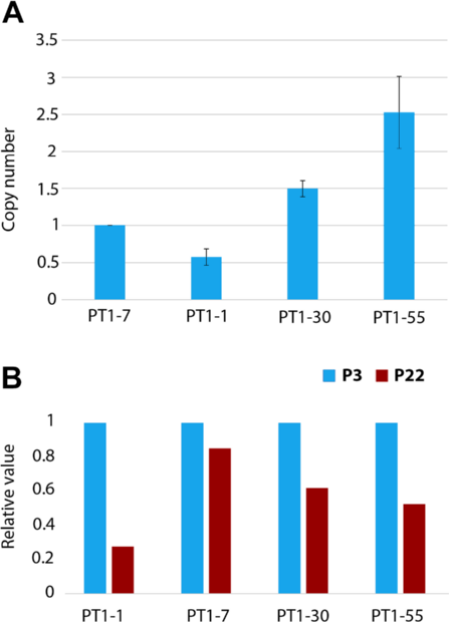
Transgene copy number and stability study in recombinant PT1-CHO cell lines. **a** Copy number of integrated transgenes was determined by quantitative PCR (qPCR) using primers on the light chain cDNA of PT1 construct at an early passage (P5). PT1-7 was used as a calibrator with a known copy number of one which was previously determined by Southern blot hybridization (data not shown). Samples were measured in triplicates. PT1-30 and PT1-55 revealed more than one copy of the transgene. Data represent means and standard error of the means (SEM) of *n* = 3 measurements. **b** A stability study was initiated with a relative value of 1 as a starting titer value. During the study, all clones showed instability to various degrees. In the most unstable clone PT1-1, a drop of productivity to 0.2 was measured whereas PT1-7 showed a loss of titer to only 0.8.

In addition, using a pair of primers specific to the light chain cDNA sequence on the PT1 construct, the copy numbers of integrated transgenes were determined by qPCR. PT1-7, with a copy number of 1 determined previously by Southern blot hybridization, served as a calibrator. The copy number of integrated transgenes ranged from about 0.5–3 copies, with PT1-55 exhibiting the highest copy number (Fig. 3a). Moreover, the PT1-CHO cell lines differed in the degree or the loss thereof of recombinant protein expression during stability studies over 22 passages (Fig. 3b, Additional file 1: Figure S1). PT1-1 was the least productive, whereas PT1-7 showed the most stable expression. Although cultivated under selection pressure (+ hygromycin), all four cell lines exhibited loss of productivity during long-term culture. Nevertheless, since two of the PT1-CHO clones with transgene copy number of about 1 still showed productivity after 22 passages (PT1-1, PT1-7), we presumed that loss of copy number could not be the sole reason for the loss of productivity. In other words, if the loss of productivity was due to a loss in transgene copy number, these clones would exhibit zero copy, which in turn implies null productivity.

### Recombinant mRNA expression

**Fig. 4:**
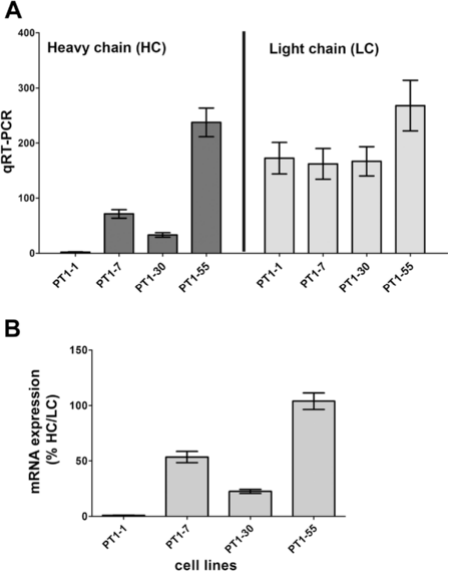
Recombinant mRNA expression in four different PT1-CHO cell lines: **a** as measured by qRT-PCR using heavy chain (HC) and light chain (LC) primers; and **b** as the percentage of HC/LC qRT-PCR values. qRT-PCR results were obtained by absolute quantification standard curve method and given as the average of *n* = 8 independent time point measurements done at several passages (P56 to P72). Two-way ANOVA was significant (***P < 0.0001); Mann – Whitney *U* test (two-tailed) for PT1-1 vs. PT1-7, PT1-30, or PT1-55) was also significant (***P = 0.0002). Data represent means and standard error of the means (SEM) of *n* = 8 independent measurements.

Because the loss of recombinant protein expression in the PT1-CHO cell lines could primarily reflect the loss of recombinant mRNA expression, we measured the mRNA expression of each cell line by qRT-PCR in various passages (i.e. P49 to P73) throughout the study. We designed PCR primers along the sequences encoding the heavy and light chains contained in the plasmid-expression-vector construct (PT1), carried out qRT-PCR for heavy and light chains using mRNA isolated from different time points and quantified recombinant mRNA expression of each (as measured by the heavy or light chain primers alone, or expressed as percentage of heavy chain over light chain qRT-PCR products) (Fig. 4, Additional file 2: Figure S2). We found significant differences in recombinant mRNA among the PT1-CHO cell lines either on the basis of heavy chain, light chain, or percentage heavy/light chain (*n* = 8, 2-way ANOVA, ***P < 0.0001). A difference with respect to light chain expression due to time point of measurements was also detected (*P = 0.0184), but not for the heavy chain. Overall, the highest expression was obtained for PT1-55, then PT1-7 and PT1-30, the lowest for PT1-1, and this relationship remained essentially constant, even in a total of *n* = 14 mRNA expression measurements (data not shown). We can deduce from this result that the poor protein productivity for PT1-1 was associated with the negligible recombinant mRNA expression of this subclone.

### DNA methylation

To determine whether DNA methylation impacts long-term gene-silencing in PT1-CHO cell lines, we next interrogated the human eukaryotic translation elongation factor 1 alpha 1 (*EEF1A1*) promoter contained in the PT1 expression vector. Using bioinformatic tools (see Methods), we annotated the 1261-bp *EEF1A1* promoter and identified two CpG islands in the promoter region (Additional file 3: Figure S3A). We designed PCR primers to analyze by bisulfite sequencing a 231-bp fragment encompassing 18 CpG sites on the CpG island nearest the transcription start site (TSS) in the PT1-CHO cell lines (Additional file 3: Figure S3B, C). Specifically, to perform DNA methylation analysis, we bisulfite-treated the total genomic DNA isolated from the PT1-CHO cell lines converting unmethylated cytosines into uracil, while methylated cytosines remain unchanged. During PCR amplification, uracils are read by DNA polymerase as thymine. Methylation state can then be determined by sequencing of the PCR product from bisulfite-modified DNA in comparison with the original sequence. Direct sequencing of amplified PCR fragments from genomic DNA isolated at high passage (P49) revealed low methylation in the analyzed 18 CpG sites of the *EEF1A1* promoter region in the four PT1-CHO cell lines (data not shown). Cloning of the PCR fragments and sequencing of clones to enable analysis of single molecules also confirmed low methylation, i.e. highest was 6.25 % found in PT1-1 (presented together with the CpG methyltransferase *M.Sss*I chromatin maps, Additional file 4: Figure S5B).

**Fig. 5:**
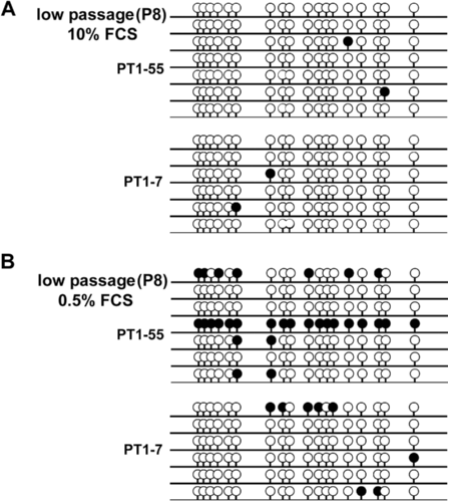
DNA methylation analysis along the *EEF1A1* promoter region at low passage (P8) but different FCS concentration 10 % (a: upper panel)  vs. 0.5 % FCS (b: lower panel) for cell lines PT1-7 vs. PT1-55. Methylated CpGs (*filled lollipops*), unmethylated CpGs (*unfilled lollipops*).

In contrast, we obtained different results when we compared the methylation patterns in the cell lines PT1-7 and PT1-55 at low passage (P8), but with reduced FCS (0.5 % instead of 10 %) in the culture medium. Thus, we observed higher methylation with 0.5 % FCS than 10 % FCS (Fig. 5), where several CpGs exhibited more than 50 % methylation level after direct bisulfite sequencing (data not shown). To verify whether CpG methylation was indeed solely due to the FCS concentration rather than passage number, we investigated the *EEF1A1* promoter region in a different vector in CHO cells at low (P2) and high passage (P22) at 10 % FCS, and under adherent culture conditions. Unlike the PT1 expression vector in which there are three copies of the *EEF1A1* promoter, there is only one promoter copy in the VT2 vector (not shown). Under these conditions, we observed more CpG methylation in VT2-CHO cell lines at late than at early passage (Additional file 5: Figure S4). Altogether, these results imply plasticity of epigenetic responses owing to different culture environments.

### Single-molecule chromatin mapping

**Fig. 6:**
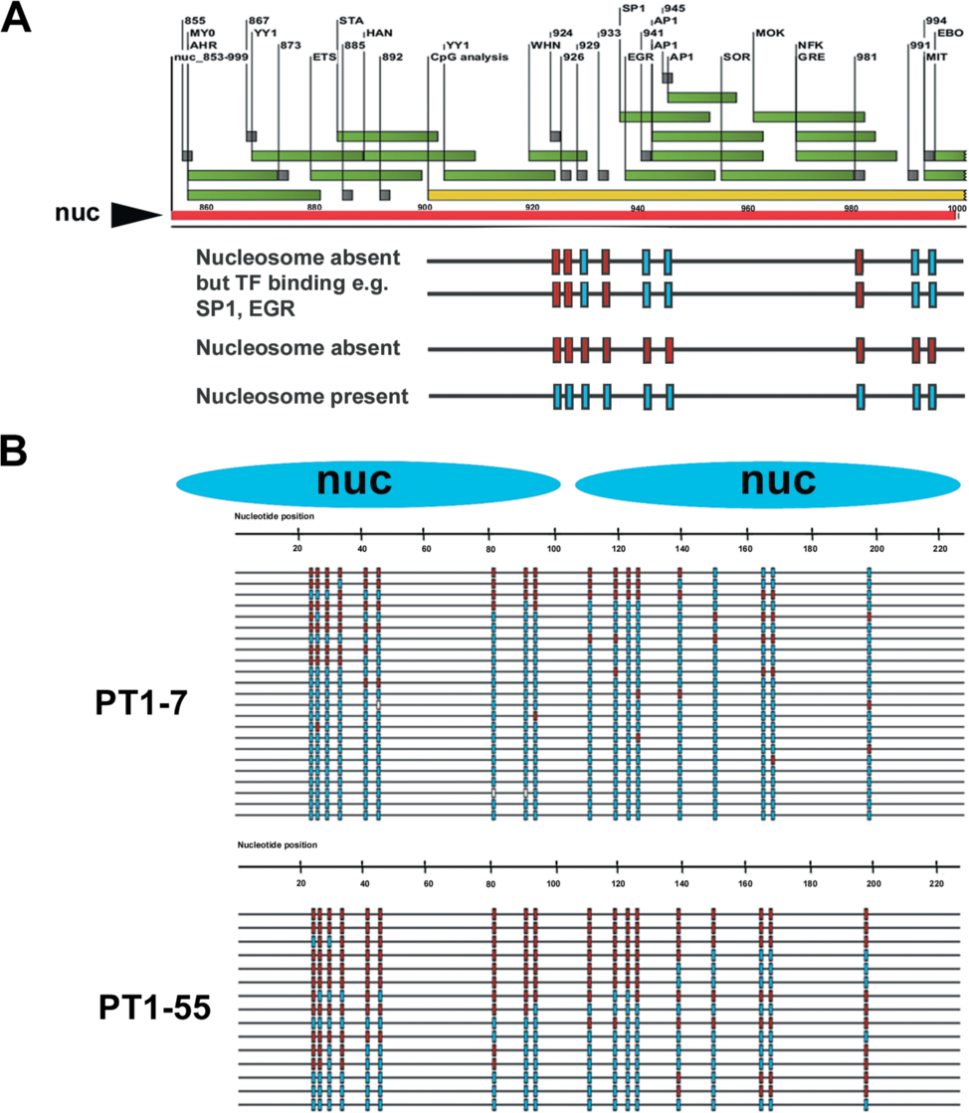
Single-molecule chromatin mapping with the CpG-specific DNA methyltransferase *M.Sss*I on the *EEF1A1* promoter in PT1-CHO cell lines. **a** Schematic annotation of a predicted nucleosome (designated as Nuc 853) with putative transcription factor binding sites (*green*, *rectangle boxes*), and CpGs (*gray*, *square boxes*). Below panels are representative *M.Sss*I maps and interpretation obtained in PT1-55. Methylated (= unprotected CpGs, *red*); Unmethylated (= protected CpGs, *blue*); white squares are missing or unclear values. **b** The analyzed nucleosomes nearest the TSS and *M.Sss*I maps showing more protected CpGs in PT1-7 than in PT1-55.

Since our data discounted a major role for DNA methylation in the repression of recombinant mRNA in the four PT1-CHO cell lines, we turned to investigating the possible contribution of the chromatin environment. We used a single-molecule footprinting strategy that reveals chromatin structure after treating nuclei with bacterial CpG-specific DNA methyltransferase (*M.Sss*I) and subsequent bisulfite sequencing of individual progeny DNA molecules [21–23]. Essentially, CpGs are methylated by *M.Sss*I unless the CpGs are blocked (or protected) by nucleosomes or DNA-binding proteins. Specific footprints can then be revealed contingent upon nucleosome positions and transcription factor binding sites on promoters (see Fig. 6a). In this regard, nucleosome localization is defined as a region of about 147-bp inaccessible to *M.Sss*I.

To facilitate correlation of *M.Sss*I chromatin maps to recombinant mRNA expression in the PT1-CHO lines, we first predicted nucleosome positions and putative transcription factor binding sites along the *EEF1A1* promoter using bioinformatic tools (described in Methods). For prediction, we used the 1261-bp *EEF1A1* promoter sequences, and analyzed the two predicted nucleosomes towards and nearest the transcription start site (TSS). For ease of scoring, these two nucleosomes were arbitrarily designated Nuc 853 (nt 853–999) and Nuc 1008 (nt 1008–1154). We next isolated chromatin from the PT1-CHO cell lines at high passage (P49), followed by a brief treatment with *M.Sss*I and genomic DNA isolation. Subsequently, we undertook bisulfite sequencing of several clones from each cell line interrogating 18 CpG sites within two predicted nucleosomes nearest the TSS, and the same sites earlier analyzed during DNA methylation analysis. Control estimates of the methylation efficiency of *M.Sss*I on the *EEF1A1* promoter obtained from ‘naked’ genomic DNA of two PT1-CHO cell lines yielded average levels of 98 % (Additional file 4: Figure S5A).

Initially, we undertook *M.Sss*I chromatin mapping with PT1-7 and PT1-55 whose results already implied a correlation with recombinant mRNA expression, recalling that recombinant mRNA expression was higher in PT1-55 than PT1-7. Indeed, the *M.Sss*I chromatin maps showed higher nucleosome occupancy (i.e. stretches of protected or unmethylated CpGs) in PT1-7 than in PT1-55 (Fig. 6b). These initial findings were confirmed after *M.Sss*I mapping involving all the four PT1-CHO cell lines which showed that nucleosome occupancy correlated well with recombinant mRNA expression (Additional file 4: Figure S5B, C). The occupancy of the nucleosome nearest the TSS (Nuc 1008) appeared most predictive, with the least productive lines (PT1-1 and PT-30) garnering the highest scores. Taken together, these results show tighter chromatin condition for PT1-1 and PT1-30 accompanying reduced mRNA expression. On the other hand, an open chromatin condition for PT1-7 and PT1-55 partnered higher expression. Nonetheless, nucleosome occupancy ranged from 62.50 to 86.67 % in these PT1-CHO cell lines which had undergone long-term culture.

### Chromatin immunoprecipitation (ChIP)

The encouraging results obtained with single-molecule mapping with *M.Sss*I, prompted further investigation of the role of chromatin structure along the *EEF1A1* promoter underlying recombinant mRNA expression and eventually protein productivity in the PT1-CHO cell lines. We carried out chromatin immunoprecipitation (ChIP), which is used to map proteins such as histones, transcription factors, and other chromatin-modifying complexes associated with specific regions of the genome. Briefly, chromatin is isolated, fragmented, and immunoprecipitated with antibodies specific to the protein or modification of interest. The purified ChIP-enriched DNA is then analyzed by quantitative-PCR, microarray technology, or sequencing [24–26]. Specifically, we performed ChIP using native chromatin (N-ChIP) fragmented by enzymatic digestion to nucleosomal resolution (150–200 bp), and antibodies against a canonical histone (H2A), two histone variants (H2A.Z, H3.3) and four histone modifications (H3K4me3, H3K27me3, H3K9ac, H3K9me3). ChIP with normal rabbit IgG was used as a control. In addition, we designed qPCR primers to amplify within the nucleosome core, borders, or fragments spanning the two nucleosomes described and analyzed earlier in the *M.Sss*I chromatin mapping. We quantified the ChIP DNA and input DNA before performing qPCR, and then normalized results using percentage input relative to the canonical histone H2A. We then correlated the different ChIP enrichments in chromatin isolated at high passages (P52 – P70) to the respective recombinant mRNA expression of the four PT1-CHO cell lines.

**Fig. 7:**
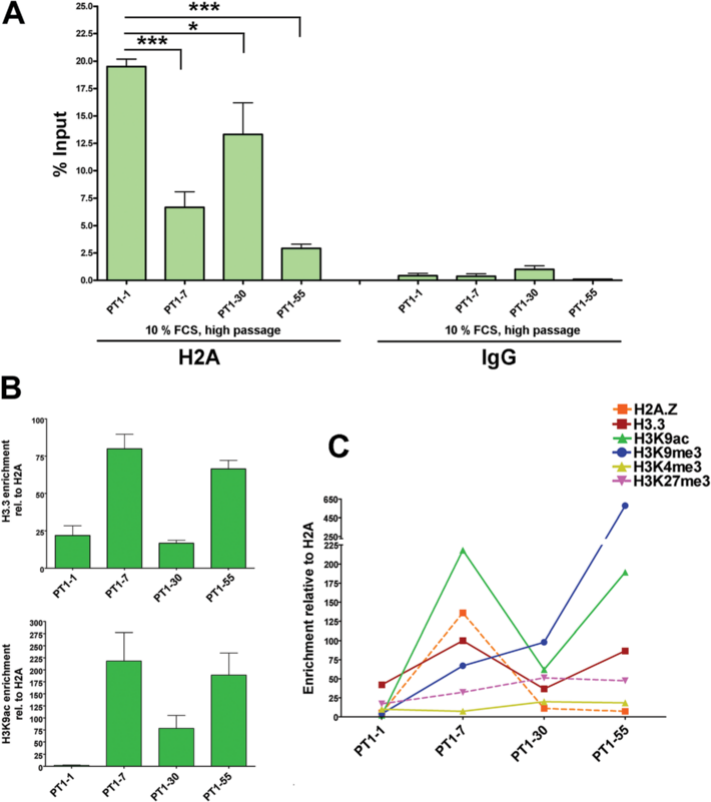
Chromatin marks associated with recombinant mRNA expression in PT1-CHO cell lines. **a** ChIP with H2A indicating higher nucleosome occupancy correlating with lower recombinant mRNA expression. Results are presented as percentage input calculated from Ct values, and the means and SEM of *n* = 12 independent experiments involving four primer pairs along two predicted nucleosome positions in the *EEF1A1* promoter region. Also shown are the qPCR values obtained in the four primer pairs for IgG, which served as a control for the ChIP experiment. Statistical significance (*, ***) at *P* < 0.05, unpaired *t* test, one-tailed. **b** H3.3 and H3K9ac enrichments relative to H2A. **c** A summary of ChIP enrichments for all the analyzed histone variants (H2A.Z, H3.3) and histone modifications (H3K4me3, H3K27me3, H3K9ac, H3K9me3). Data represent means and standard error of the means (SEM) of *n* = 3 independent experiments and in chromatin isolated at different passages (P52 – P70).

That a tight chromatin conformation was associated with repression of recombinant mRNA expression or vice versa was confirmed by the ChIP results obtained with the canonical H2A antibody (Fig. 7a, Additional file 6: Figure S6A). Specifically, we performed ChIP with H2A alone in all the four PT1-CHO cell lines. H2A was included as control for histone quality in all subsequent ChIP experiments with histone variants and histone modifications. Thus, there were a total of *n* = 12 ChIP experiments with H2A. ChIP-PCR was undertaken using the four primer pairs on two predicted nucleosome positions along the human *EEF1A1* promoter region. We thus showed significant differences in H2A enrichments (i.e. H2A nucleosome occupancy) among the four cell lines (two-way ANOVA, **P = 0.0070). Differences owing to the qPCR primer pair used (i.e. nucleosome) were not significant. Notably, we observed higher H2A enrichments for PT1-1 and PT1-30 than PT1-7 and PT1-55, with PT1-1 garnering the highest while PT1-55 was the lowest (e. g. *t*-test ***P < 0.0001 for PT1-1 vs. PT1-7 or PT1-55). Altogether, H2A enrichment negatively correlated with the recombinant mRNA expression, consistent with the findings previously obtained with the *M.Sss*I chromatin mapping.

ChIP enrichments of histone variants and histone modifications are given as percentage input DNA and/or further normalized relative to the histone control H2A (Fig. 7, Additional file 6: Figure S6) in which results could vary, especially regarding histone modifications (see e.g. Additional file 6: Figure S6C, E, F, G). The normalization with an invariant histone (e.g. H2A) is assumed to correct for differences in ChIP signals caused by differences in nucleosome density [27]. Nonetheless, on the basis of ChIP enrichments relative to H2A, we found significant differences among the PT1-CHO cell lines with respect to the histone variants (H2A.Z, H3.3.) and the histone modifications (H3K4me3, H3K27me3, H3K9ac, H3K9me3). For instance, enrichments of H3.3 and H3K9ac (i.e. nucleosome occupancy) were highly significant (two-way ANOVA, ***P < 0.0001 for H3.3, **P = 0.0027 for H3K9ac) and correlated positively with recombinant mRNA expression (Fig. 7b). No significant differences were detected of enrichments owing to the ChIP-qPCR primers used (i.e. specific nucleosome). Overall, the cell lines with the least recombinant mRNA expression (PT1-1, PT1-30) also displayed the least ChIP enrichments or vice versa (Fig. 7c). Furthermore, PT1-30 obtained the highest level for H3K27me3 (see also Additional file 6: Figure S6B). As for the cell lines with highest expression, PT1-7 displayed the highest enrichment for H2A.Z, and PT1-55 for H3K9me3. Among the histone variants and modifications analyzed, there was a low level of H3K4me3 in all the PT1-CHO cell lines.

### Formaldehyde-assisted isolation of regulatory elements (FAIRE) analysis

**Fig. 8:**
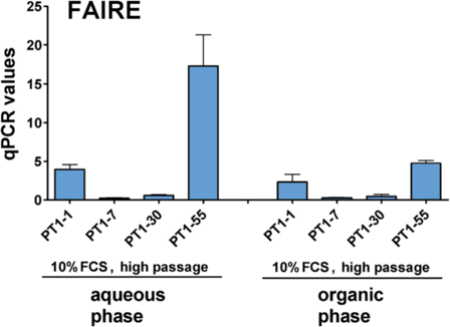
FAIRE-enrichment (DNA not bound by protein, aqueous phase) showing highest in PT1-55 as measured by qPCR. The recovered fragments in the corresponding organic phase are also shown. Primers used are given in Table 2. Data represent means and standard error of the means (SEM) of *n* = 3 independent experiments and in chromatin isolated at different passages (P55, P72, P73).

The *M.Sss*I mapping and ChIP with H2A suggested a more permissive chromatin, i.e. lesser nucleosome occupancy for PT1-55 than the other three PT1-CHO cell lines, which was associated with higher recombinant mRNA expression. To determine chromatin openness for PT1-55, we adopted a strategy using FAIRE (formaldehyde-assisted isolation of regulatory elements) coupled with qPCR with the same primers used in ChIP experiments. FAIRE identifies nucleosome-depleted regions; i.e. regions (= regulatory elements) bound by transcription factors or other regulatory proteins [28]. Essentially, the technique involves crosslinking of chromatin with formaldehyde followed by sonication, phenol-chloroform extraction, and DNA isolation. DNA fragments recovered from the aqueous phase (i.e. DNA not bound by protein) are then analyzed by PCR, microarrays, or next-generation sequencing. We found significant differences among the PT1-CHO cell lines concerning FAIRE enrichment (2-way ANOVA *P = 0.0114), but not on specific primers used. Crucially, PT1-55 exhibited the highest FAIRE-enrichment levels (Fig. 8).

### Chromatin signatures in PT1-CHO cell lines

Multiple approaches, i.e. DNA methylation analysis, single-molecule chromatin mapping with *M*.SssI, ChIP with different histones and FAIRE on the *EEF1A1* promoter contained in the expression vector integrated in the CHO genome revealed that each PT1-CHO cell line displayed a specific epigenetic signature or chromatin marks predictive of recombinant mRNA expression (Table 1). For instance, the cell line (PT1-1) with the lowest recombinant mRNA expression had the highest nucleosome occupancy and displayed the least enrichments of histone marks particularly those associated with active transcription. On the other hand, the cell line PT1-55 which showed the highest recombinant mRNA expression also exhibited the highest FAIRE enrichments, and the greatest histone modifications H3K9ac and H3K9me3 which mark active promoter regions. Furthermore, cell line PT1-7 with the second highest recombinant mRNA transcription exhibited the highest enrichments of the histone variants H3.3 and H2A.Z, and the histone modification H3K9ac. Altogether, these results suggest that chromatin structure along the *EEF1A1* promoter region is predictive of recombinant mRNA expression in the analyzed PT1-CHO cell lines and culture conditions, and in turn might have contributed to the eventual loss of recombinant protein expression after long-term culture.

**Table 1:** Summary of the different epigenetic results and correlation to recombinant mRNA expression in the PT1-CHO cell lines

Cell lines	mRNA HC/LC (%)	ChIP enrichment (% Input)							FAIRE	*M.SssI* map (% nucleosome occupancy)		% Methylation
		H2A	H3.3	H2A.Z	H3K9ac	H3K9me3	H3K4me3	H3K27me3		Nuc 853	Nuc 1008	
PT1-1	1.00	**19.50**	22.00	5.26	2.17	3.90	8.04	17.20	46.90	60.00	**86.67**	**6.25**
PT1-7	53.50	6.67	**79.94**	**135.97**	**218.15**	66.96	5.41	32.23	2.60	60.67	66.67	0.00
PT1-30	22.36	13.31	16.73	11.13	61.99	97.82	**17.90**	**51.18**	6.00	73.33	80.00	2.08
PT1-55	104.00	2.93	66.45	7.29	189.00	**577.78**	16.40	47.40	**207.50**	**87.50**	62.50	0.71
Pearson correlation		−0.96	0.76	0.12	0.83	0.91	0.30	0.54	0.77	0.76	−0.95	−0.75

mRNA expression based on ratio qRT-PCR product of heavy-chain primers to light-chain primers (%); ChIP enrichments for histone variants and modifications relative of H2A; FAIRE enrichments (qPCR values using primers described in Materials and Methods); highest score bold and underlined.

## Discussion

Epigenetic silencing of the recombinant gene can be listed among the prime causes leading to reduced recombinant protein production. Thus, we analyzed epigenetic modifications affecting chromatin structure that are associated with decreases or loss of recombinant mRNA expression during cell line development for recombinant protein production. Using a variety of approaches, we investigated the DNA methylation pattern and chromatin landscape around the human *EEF1A1* promoter sequence contained in the expression vector (PT1) used for antibody production, and which was integrated into the CHO genome. We analyzed four PT1-CHO cell lines which differed in the loss-of-protein expression seen after high passage (> P35) in adherent condition supplemented with 10 % FCS in the culture medium. We found that epigenetic signatures in the PT1-CHO cell lines correlated highly with recombinant mRNA expression. Furthermore, the lowest-producing cell line exhibited chromatin marks suggestive of tight chromatin, while the highest-producing line showed marks associated with open chromatin. Our results thus demonstrate that the mapping of chromatin structures can be useful in CHO cell line development and metabolic engineering.

Previous studies have shown that DNA promoter hypermethylation can contribute to instability among recombinant CHO cell lines. For instance, CpG sites within the human cytomegalovirus major immediate early promoter/enhancer (hCMV-MIE) are frequently methylated in unstable antibody-producing CHO cell lines [18, 19]. We also investigated the DNA methylation pattern around the human *EEF1A1* promoter, but none of the PT1-CHO cell lines under the condition of 10 % FCS and high passage exhibited significant increases in CpG methylation to suggest that DNA methylation alone underlay reduced production. Nonetheless, our results also implied culture-context-dependent methylation of the *EEF1A1* promoter. Higher methylation became evident in the same CHO cell line even at low passage if the FCS concentration of the culture medium was reduced from 10 to 0.5 %. Furthermore, when present in another vector construct, methylation along the *EEF1A1* promoter in CHO cell lines was enhanced at higher passage numbers (see Results and Additional file 5: Figure S4).

The chromatin mapping with the DNA methyltransferase *M.Sss*I (a CpG methylase) provided visualization of nucleosome occupancy on single *EEF1A1* promoter molecules, which correlated well with the recombinant mRNA expression, i.e. higher nucleosome occupancy meant lower mRNA expression. Nonetheless, there was high nucleosome occupancy, ranging from 62.5 to 86.7 %, in the PT1-CHO cell lines which had undergone long-term culture. Recently, a similar approach of nucleosome mapping in yeast *PHO5* promoter with *M.CviP*I (a GpC methylase) in single cells revealed significant heterogeneity of nucleosome configurations within a population [29]. The cell-to-cell variation in nucleosome positions and shifts in nucleosome positioning correlated with changes in gene expression. Such underlying complexity of nucleosome positioning can contribute to the flexibility and heterogeneity of gene expression. Thus, mapping of nucleosomes using DNA methyltransferases could facilitate weeding out unstable CHO cell clones early when screening for stable-expressing cell lines. Nevertheless, proper analytical methods and controls must be observed when performing nucleosome mapping experiments especially in combination with next-generation technologies [30].

Histones form the protein core of chromatin around which DNA is wrapped. Various modifications to histones play a key role in epigenetic control of cellular activity [31]. Histone modifications and crosstalk can dictate the structure of chromatin as well as its functions in transcription, replication, and DNA repair. We performed ChIP assays on different histones (canonical, histone variants and histone modifications) to elucidate their role in recombinant protein abatement in the PT1-CHO cell lines. Indeed, a critical role of acetylated histone H3 (H3ac) in the stability of recombinant protein production has been shown previously [20]. The decline of recombinant antibody production during long-term culture was attributable to a 48–53 % decrease in recombinant mRNA levels without significant loss of recombinant gene copies, but accompanied by an approximately 45 % decrease in H3ac. Our ChIP results on the analyzed PT1-CHO cell lines showed a strong correlation of histone enrichments with transcriptional and protein status. For instance, the level of H2A-nucleosome occupancy was found highly predictive of chromatin structure along the *EEF1A1* promoter in affecting recombinant mRNA expression and productivity in the PT1-CHO cell lines.

Certain histone variants and histone post-translational modifications (PTMs) have been considered variously as chromatin marks for transcriptional activation or repression. For example, the histone modification H3K4me3 is often associated with transcriptional activation [32], and this PTM was not enriched in the PT1-CHO cell lines analyzed after long-term cell culture. Interestingly, the cell line PT1-7 which also showed high recombinant mRNA transcription and the most stable protein expression, was marked by enrichments of positive chromatin marks: i.e. histone variants H2A.Z, H3.3, and the histone modification H3K9ac (see Table 1). Deposition of H2A.Z and H3.3 onto nucleosomes is generally associated with active transcription [33]. Nucleosomes containing double variants H2A.Z and H3.3 are found in ‘nucleosome-free’ regions of active promoters in human cells [34]. Moreover, acetylation of specific lysine residues of H3 (e.g. H3K9ac) in promoter regions also correlates with gene activation [35].

A well-known PTM and a key determinant of complex chromatin states is the methylation of histone lysine residues. Histone methylation (mono-, di-, tri-) of lysine residues is catalyzed by SET-domain containing proteins and plays a critical role in the regulation of gene expression, cell cycle, genome stability, and nuclear architecture (see reviews [36–38]). Histone lysine methylation is a marker of both transcriptionally active and inactive chromatin, depending on the residue methylated and the degree of methylation. For instance, methylation of H3K4 is associated with active chromatin, while methylation of H3K27 and H3K9 are generally hallmarks of condensed chromatin, and thus involved in gene silencing [38]. But active and inactive chromatin marks can colocalize in bivalent domains associated with transcriptional repression such as in pluripotent embryonic stem (ES) cells and restricted trophoblast stem (TS) cells to poise differentiation genes before activation or to stably repress genes [39]. Our ChIP results showed that PT1-30 and PT1-55 exhibited higher levels for H3K9me3 which may be explained by their integration sites within repetitive regions (i.e. centromeric for PT1-30, while telomeric for PT1-55) in the CHO genome as shown by FISH. Indeed, the enzymes (SUV39H1/2) mediating the bulk of H3K9 trimethylation preferentially localize to pericentric heterochromatin and telomeres (see [37]). Similarly, PT1-30 had the highest H3K27me3 enrichments which may be also attributed to its centromeric integration. Combined with emerging new technologies, the field of epigenetics will surely gain a prominent role in recombinant protein production. The advent of ‘omics’ coupled with next-generation technologies and the sequencing of the CHO genome [40, 41] can lead to enormous progress towards insights and innovations in CHO cell line development and metabolic engineering [42, 43]. Furthermore, a CHO-specific microarray can now be used for the analysis of differential genome-wide CpG methylation after butyrate supplementation, which is known to enhance cell-specific productivities in CHO cells [44]. At the same time, a tremendous amount of information is accumulating in the field of epigenetics, and on the mechanisms underlying chromatin structure and function [45, 46], e.g., the chromatin-based silencing mechanisms such as the polycomb system and heterochromatin formation [47]. Furthermore, an array of approaches is available to understand epigenetic inheritance and to predict functional gene expression at the genome or single-cell level [48, 49].

## Conclusions

After carrying out DNA methylation analysis, followed by single-molecule chromatin mapping with *M*.SssI, ChIP with different histones and FAIRE on the *EEF1A1* promoter contained in an expression vector integrated into the CHO genome, we found that each PT1-CHO cell line displayed a distinct epigenetic signature or chromatin marks indicative of recombinant mRNA expression. Altogether, these results suggest that the chromatin structure along the *EEF1A1* promoter region can be predictive of recombinant mRNA expression in the PT1-CHO cell lines analyzed and together with culture conditions might contribute to the eventual loss of recombinant protein expression after long-term culture. These findings provide insights on how unstable CHO cell clones could be weeded out early in the generation process of stable-expressing cell clones and protein production might be better assured.

## Methods

### Establishment of PT1 cell lines

Briefly, adherent CHO-K1 cells (DSMZ, ACC110) were transfected with a vector carrying cDNA of the heavy and light chain of a murine IgG2a antibody. After selection of transfected cells with 500 μg/mL Hygromycin B, a limiting dilution was performed to obtain the clonal cell lines, namely: PT1-1, PT1-7, PT1-30, and PT1-55.

### Fluorescence in situ hybridization (FISH)

FISH was performed to determine integration sites of the plasmid-expression-vector construct (PT1) in the CHO genome. Cytogenetic harvesting and FISH were performed on short-term cultures (P10) according to protocols described previously [50, 51]. Metaphase-enriched subconfluent cultures were prepared for cytogenetic analysis as described previously [50]. FISH probes were labeled by nick translation with fluor-dUTPs (Dyomics, Jena, Germany). Hybridizations were performed overnight. Slides were washed in 0.5X SSC and counterstained with 50 ng/mL DAPI (4′,6-diamidino-2-phenylindoledihydrochloride) in Vectashield antifade mounting medium (Alexis, Gruenberg, Germany), visualized using a Zeiss Axioimager microscope via a 100× alpha-Planapochromatic (Zeiss, Jena, Germany) configured to a HiSKY image analysis system (Applied Spectral Imaging, Neckarhausen, Germany).

### Cytogenetic analysis

We performed cytogenetic analysis on PT1-CHO cell lines using standard procedures. PT1-CHO cells (0.25 × 10^6^) were grown at 37 °C in T25 culture flasks containing 5 mL culture medium (Ham’s F12, Gibco, Thermo-Fischer, Schwerte, Germany) supplemented with 8 mM GlutaMAX, 10 % FCS (Sigma-Aldrich, Taufkirchen, Germany) and 50 μg/mL Hygromycin B (Sigma-Aldrich). After 48 h, 150 μL of 10 μg/mL colcemid (Sigma-Aldrich) was added for 2 h. For hypotonic treatment, cells were detached enzymatically with trypsin-EDTA (Thermo Fischer, Schwerte, Germany). After washing, 5 mL pre-warmed 1 % sodium citrate was added and incubated for 10 min at 37 °C. Cells were fixed in 3 parts methanol : 1 part glacial acetic acid, and dropped onto ice-cold pre-cleaned microscope slides. Metaphase spreads were stained with DAPI for fluorescence microscopy. The chromosome numbers at passage P55 were determined from *n* =100 metaphases per cell line.

To determine the frequencies for micronucleus (MN), nuclear bud (NBUD), ana/telophase aberrations, and chromatin condensation/fragmentation, PT1-CHO cells (6.6 × 10^4^) were grown in single-well chamber slides (Nunc-LabTek) in 3 mL medium as described above for 24 h at 37 °C. Cells were fixed in 3 parts methanol : 1 part glacial acetic acid and stained with DAPI for microscopy. Per cell line, *n* = 4000 cells were scored for MN, NBUD, and chromatin condensation/fragmentation, while mitotic aberrations determined in *n* = 100 ana/telophases. Results are presented as the mean of two passages (P55 and P72).

### Copy number determination

The copy numbers of the transgene were determined by qPCR on the light chain (PT1-LC-Fwd-2/ PT1-LC-Rev-2), and a reference gene *Vezt* (Vezt-305-up/ Vezt-305-dw) in host line, at an early passage number (P5). Oligonucleotide primer sequences are given in Table 2. Copy numbers were expressed as ratios of individual copy numbers relative to a calibrator. PT1-7 was used as a calibrator with a known copy number of 1, which was previously determined with Southern blot hybridization (data not shown). Copy numbers were determined in triplicate.

**Table 2:** Primers used in the analysis of PT1-CHO cell lines

Primer designation	Type of analysis	Primer sequence (5′-3′)	Amplicon size (bp)
PTl-HC-Fwd	qRT-PCR	GTGAAGGGCCGATTCACTAT	179
PTl-HC-Rev		TTGGCTGAGGAGACTGTGAC	
PTl-LC-Fwd	qRT-PCR	GGCACACGGTATTCTCTCAA	192
PTl-LC-Rev		TGAGGCACCTCCAGATGTTA	
PTl-LC-Fwd-2	qPCR	ACCAACCGTATCCATCTTCC	125
PTl-LC-Rev-2		AACTGTTCAGGACGCCATT	
Vezt_305_up	qPCR	TGAACTTGAAAGCTCGTTTG	134
Vezt_305_dw		CTCCGGAGCAGTTTTATCCAC	
EEF1A1-B5-Fwd	Bisulfite sequencing	TTGTTGTAGGGAGTTTAAAATGGAG	231
EEFlAl-BS-Rev		TCCACCCACTCAATATAAAAAAACT	
EEF1A1-Nuc853-Fwd	ChIP, FAIRE	AGTTGCGTGAGCGGAAAGAT	111
EEF1A1-Nuc853-Rev		CCTTTGTGTGGGTGACT	
EEF1A1-Nucl008-Fwd	ChIP, FAIRE	GATTAGTTCTCGAGCTTTTGGAGT	102
EEF1A1-Nucl008-Rev		CCTAACTTCAGTCTCCACCCACT	
EEF1A1-Nuc853-1008-Fwd	ChIP, FAIRE	GTGAGTCACCCACACAAAGG	107
EEF1A1-Nuc853-1008-Rev		GCTCGAGAACTAATCGAGGTG	
EEF1A1-Nucl008 right-Fwd	ChIP, FAIRE	CACACTGAGTGGGTGGAGAC	107
EEFlAl-NuclOOS right-Rev		TGAGGCTTGAGAATGAACCA	

### Stability study of PT1 cell lines

For stability study of the PT1 clonal cell lines, cells were cultivated for 22 passages in Ham’s F12 medium supplemented with 6 mM L-Glutamine and 500 μg/mL Hygromycin B and passaged every 3–4 days. Every third passage, supernatants were collected for titer determination. To determine the specific productivity, seeding and harvesting cell densities were determined. Specific productivity was calculated according to equations (1) and (2):

1 $$ IVCC\ \left[{10}^9c \times h\times {L}^{-1}\right] = \frac{\left({c}_{\mathrm{XV}1}+{c}_{\mathrm{XV}0}\right)}{2} \times \left({t}_1-{t}_0\right) $$

2 $$ {q}_{\mathrm{p}}\ \left[ pg{\left(c\times d\right)}^{-1}\right]=\frac{\left({c}_{\mathrm{P}1}-{c}_{\mathrm{P}0}\right)}{(IVCC)}\times 24 $$

IVCC = Integral of the viable cell concentration

C_XV1_ = viable cell density (10^9^/L) at time point t_1_

C_XV0_ = viable cell density (10^9^/L) at time point t_0_

qP = specific productivity (pg/cell/day)

C_P1_ = Titer (μg/mL) at time point t_1_

C_P0_ = Titer (μg/mL) at time point t_0_

### Titer determination by ELISA

Microtiter plates (96-well) were coated with a goat anti-mouse antibody (cat no. GTX77320, GeneTex, Irvine, USA) and incubated with cell culture supernates overnight at 8 °C. Purified mouse IgG2a κ, isotype control (Cat no. 400201, BioLegend, San Diego, USA) served as a standard. Detection was performed after incubation with a horseradish-peroxidase conjugated goat anti-mouse IgG (Cat no. 18-511-244228, GenWay, San Diego, USA) followed by incubation with the substrate o-Phenyldiamindihydrochloride and H_2_O_2_. After the reaction was stopped with 1 M H_2_SO_4_, optical densities (OD) were measured at 490 nm with a SpectraMax 190 (Molecular Devices, Sunnyvale, USA).

### Cell culture conditions for epigenetic analysis

For epigenetic analysis, approximately 1 × 10^6^ cells from each PT1-CHO cell line were grown in T75 culture flasks as described above (see cytogenetic analysis) until about 100 % confluency (2–3 d) to obtain circa 1 × 10^7^ cells. Cells were pelleted, and washed twice with 1 mL cold PBS. Cell pellets were used immediately or flash frozen in liquid nitrogen and stored at 80 °C for subsequent analysis.

### Bioinformatic predictions

To determine epigenetic and transcriptional heterogeneity in the PT1-CHO cell lines, we used approaches and protocols, as previously described [52, 53]. We predicted nucleosome positions along the promoter sequence (1261-bp) of *EEF1A1* using bioinformatic tools such as: (1) nucleosome prediction by genomic sequence (http://genie.weizmann.ac.il/software/nucleoprediction.html) [54–56]; (2) NuPoP:Nucleosome Positioning Prediction Engine (http://nucleosome.stats.northwestern.edu/)[57]; and (3) The ICM Web (http://dna.ccs.tulane.edu/icm/) [58]. We analyzed the promoter region of *EEF1A1* for putative binding sites of transcription factors using TRANSFAC (http://www.gene-regulation.com/pub/databases.html) or MatInspector (Genomatix, https://www.genomatix.de). We annotated the *EEF1A1* promoter using CLC Workbench (www.clcbio.com/products/clc-main-workbench).

### Recombinant mRNA expression analysis

We analyzed recombinant mRNA essentially as described previously [52]. We isolated total RNA from frozen cell pellets with the RNeasy Mini kit using the recommended protocol (Qiagen, Hilden, Germany). Reverse-transcription-PCR (RT-PCR) was performed with the Omniscript RT kit according to the manufacturer’s protocol (Qiagen). qRT-PCR was undertaken on ABI 7500 Real-Time PCR System (Life Technologies). Quantification of cDNA template (50 ng/reaction) was determined using NanoDrop (PeqLab, Erlangen, Gemany). PCR reaction components and cycling variables were according to standard procedures. Recombinant mRNA expression was determined by absolute quantification using the standard curve method. A dilution series of the genomic DNA of cell line PT1-7 served as a standard. Primer sets designed for the heavy (HC) and light (LC) chain sequences contained in the PT1 vector were used for the analysis (Table 2).

### Epigenetic analyses

We performed DNA methylation analysis as previously described [52]. Briefly, genomic DNA was isolated from frozen cell pellets of PT1-CHO cell lines using Nucleo Spin Tissue (Macherey-Nagel, Düren, Germany). Bisulfite treatment of approximately 1 μg DNA per cell line was undertaken with EpiTect Bisulfite kit (Qiagen) using the manufacturer’s instructions. Determination of CpG islands and design of primers for the methylation assay were performed with MethPrimer (http://www.urogene.org/methprimer/index1.html) on the human eukaryotic translation elongation factor 1 alpha 1 (*EEF1A1*) promoter contained in the PT1 vector (Table 2). We sequenced amplified PCR fragments directly or cloned fragments before sequencing using a TOPO TA Cloning kit (Life Technologies). Sequencing was undertaken using BigDye Terminator v3.1 kit and ABI 3130XL Genetic Analyzer (Life Technologies). Sequences were analyzed with SeqMan (DNAStar Lasergene), CpGviewer (http://dna.leeds.ac.uk/cpgviewer/) or BISMA (http://services.ibc.uni-stuttgart.de/BDPC/BISMA/).

The protocols used for the various experiments on chromatin, i.e. chromatin isolation, *M.Sss*I treatment, micrococcal nuclease (MNase) digestion, CHIP, and FAIRE have been described earlier [52]. ChIP experiments on histones were done with N-ChIP, which uses native chromatin fragmented by MNase digestion, thus yielding a nucleosome-based resolution. We analyzed the ChIP DNA using quantitative PCR. We used four primer sets along the human *EEF1A1* promoter in the PT1 vector (Table 2). These primer sets were designed to amplify within the core of two putative nucleosome positions (Nuc853F/R, Nuc1008F/R), straddling (Nuc853-1008 F/R), or on the right border of a nucleosome (Nuc1008-rightF/R). DNA concentration (ChIP- and input-DNA) was determined using a NanoDrop ND-1000 spectrophotometer (PeqLab), and template DNA for each qPCR reaction was adjusted to 20 ng. The template DNA in IgG controls also consisted of 20 ng. A dilution series of the genomic DNA of cell line PT1-7 served as a calibration standard for the qPCR. For each histone, at least three independent ChIP experiments were performed. Data are given as percent input values based on 1 % of starting chromatin and Ct values. Statistical significance was carried using ANOVA and *t*-tests contained in GraphPad Prism. About 5 μg of antibody was used for each ChIP experiment. Histone antibodies were obtained from Abcam: H2A (ab18255), H2A.Z (ab18263), H3.3 (ab62642), H3K4me3 (ab1012), H3K27me3, (ab6002), H3K9me3 (ab4441), and H3K9ac3 (ab8898). The normal rabbit IgG antibody (sc-2027) used as a control was obtained from Santa Cruz Biotechnology (Heidelberg, Germany).

## Additional files

Additional file 1: Figure S1. Monitoring of recombinant protein expression. Four recombinant CHO-K1 clonal lines were subjected to a stability study for 11 weeks. During cultivation, all four PT1-CHO cell lines showed a drop in specific productivity. A severe instability was found for PT1-1. Note that at week 1, the specific productivity was lower than in week 3. This was possibly due to recovery of cells from thawing process resulting in low cell densities. Values from week 3 and 11 were used for calculation of relative values in Fig. 3b. (TIF 209 kb) Additional file 2: Figure S2. Recombinant mRNA expression in four different PT1-CHO cell lines. (A) A schematic diagram of the PT1 vector showing the location of fragments amplified by the qRT-PCR heavy chain (HC) and the light chain (LC) primers. (B) mRNA expression as measured by the HC (left panel) and by LC (right panel) primers after a two-month continuous adherent culturing and passaging (P56 to P72) in 10 % FCS. (C) mRNA expression of same samples based on percentage of the HC qRT-PCR values (B, left panel) over that of LC values (B, right panel). (TIF 753 kb) Additional file 3: Figure S3. DNA methylation analysis in the *EEF1A1* promoter region in PT1-CHO cell lines. (A) A schematic representation of the two CpG islands and bisulfite sequencing primers as identified using MethPrimer (http://www.urogene.org/methprimer/index1.html) (B) Sequence of the analyzed fragment (231-bp) and the analyzed CpGs (arrows). The last nucleotide in the 1261-bp *EEF1A1* promoter towards the transcription start site (TSS) was designated as −1. (C) Partial Sanger sequence electropherogram with methylated CpGs underlined. (TIF 2035 kb) Additional file 4: Figure S5.
*M.Sss*I chromatin mapping on the *EEF1A1* promoter in PT1-CHO cell lines. (A) Schematic annotation of the promoter region and the comparison of results in chromatin and ‘naked’ genomic DNA after *M.Sss*I treatment. In ‘naked’ genomic DNA, most CpGs are methylated, while the unmethylated ones are random. In the chromatin, stretches of unmethylated or protected CpGs are evident to suggest occupancy of a nucleosome. (B) *M.Sss*I chromatin maps of the four PT1-CHO cell lines (left panel); corresponding DNA methylation pattern (bisulfite-treated genomic DNA only), indicating endogenous methylation on the same cell lines (right panel). Methylated CpGs, red); Unmethylated CpGs, blue). (C) Nucleosome occupancy in the two analyzed nucleosomes Nuc 853 (nt 853–999) and Nuc 1008 (nt 1008–1154). (TIF 1777 kb) Additional file 5: Figure S4. DNA methylation analysis in the *EEF1A1* promoter region in VT2-CHO cell lines, at low vs. high passage. Methylated CpGs (filled lollipops), unmethylated CpGs (unfilled lollipops). (TIF 738 kb) Additional file 6: Figure S6. ChIP enrichments in PT1-CHO cell lines. (A) H2A enrichments in the different PT1-CHO cell lines and four qPCR primer pairs. The data were analyzed by two-tailed ANOVA, which found statistically significant differences in H2A enrichment (H2A nucleosome occupancy) among the cell lines (** *P* = 0.0070), but not differences resulting with the qPCR primers (nucleosome). Results are presented as % input calculated from Ct values, and the means and SEM of *n* = 12 independent experiments involving four primer pairs along two predicted nucleosome positions in the *EEF1A1* promoter region. Also shown are the qPCR values obtained in the four primer pairs for IgG, which served as control for the ChIP experiment. (B) ChIP with H3K27me3 and control IgG obtained after qPCR with four primer pairs. (C, E, F, G) ChIP with H2A.Z, H3K9me3, and control IgG obtained by % input DNA, left panels), and H2A.Z and H3K9me3 after further normalization to H2A (right panels). Data represent means and SEM of *n* = 3 independent experiments. (TIF 799 kb)

## Abbreviations

CHO: Chinese hamster ovary
ChIP: chromatin immunoprecipitation
N-ChIP: native chromatin immunoprecipitation
*EEF1A1*: human eukaryotic translation elongation factor 1 alpha 1
FISH: fluorescence in situ hybridization
qPCR: quantitative polymerase chain reaction
qRT-PCR: quantitative reverse transcription polymerase chain reaction
FAIRE: formaldehyde-assisted isolation of regulatory elements
MNase: micrococcal nuclease
FCS: fetal calf serum
