# Supplementary figures and images for: Mechanisms underlying epigenetic and transcriptional heterogeneity in Chinese hamster ovary (CHO) cell lines
Source: BMC Biotechnol. 2016 Jan 22;16:6. doi: 10.1186/s12896-016-0238-0 (PMC4722726; doi:10.1186/s12896-016-0238-0)

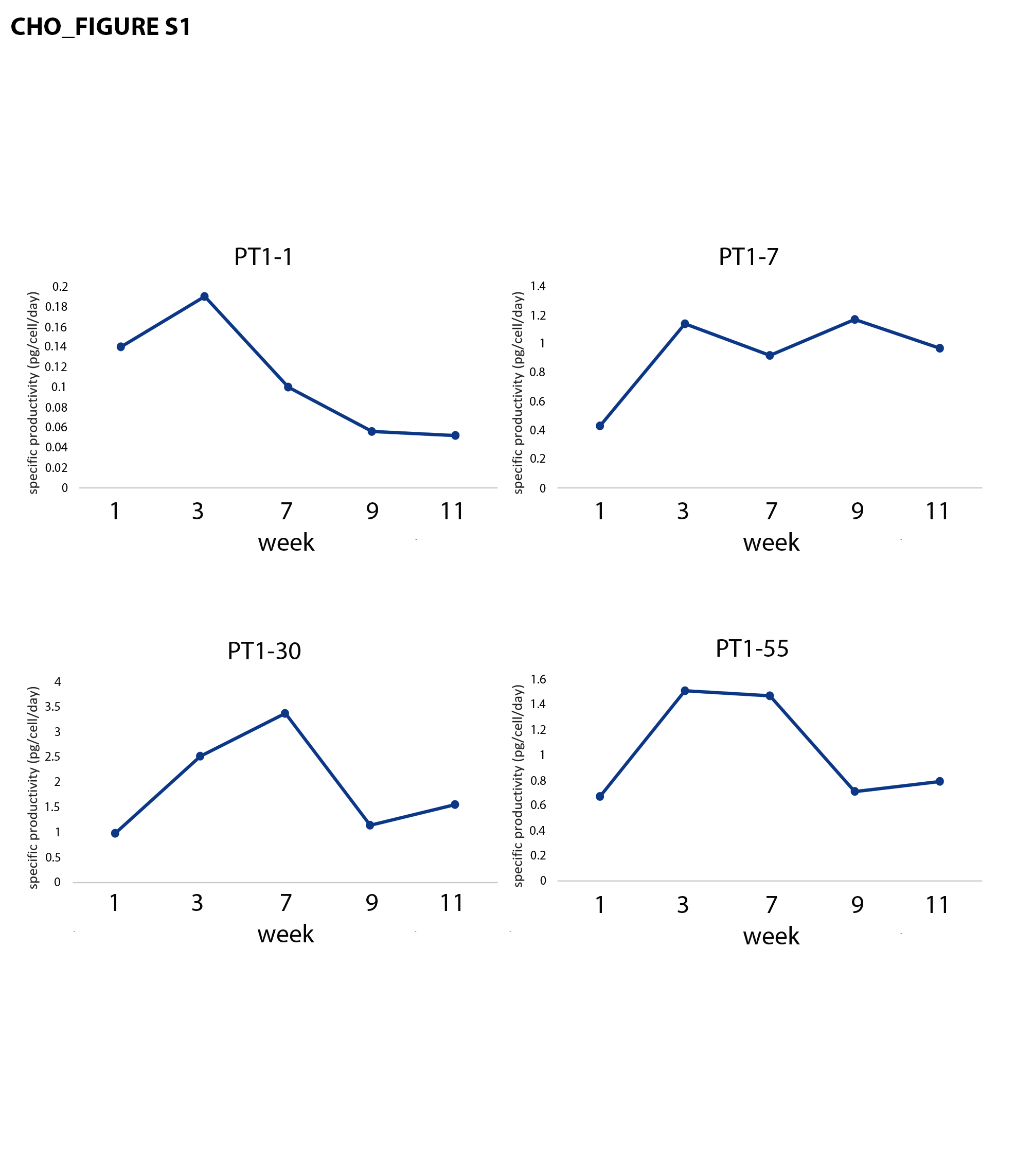

Supplement: Additional file 1: Figure S1. — Monitoring of recombinant protein expression. Four recombinant CHO-K1 clonal lines were subjected to a stability study for 11 weeks. During cultivation, all four PT1-CHO cell lines showed a drop in specific productivity. A severe instability was found for PT1-1. Note that at week 1, the specific productivity was lower than in week 3. This was possibly due to recovery of cells from thawing process resulting in low cell densities. Values from week 3 and 11 were used for calculation of relative values in Fig. 3b. (TIF 209 kb) [file 12896_2016_238_MOESM1_ESM.tif]

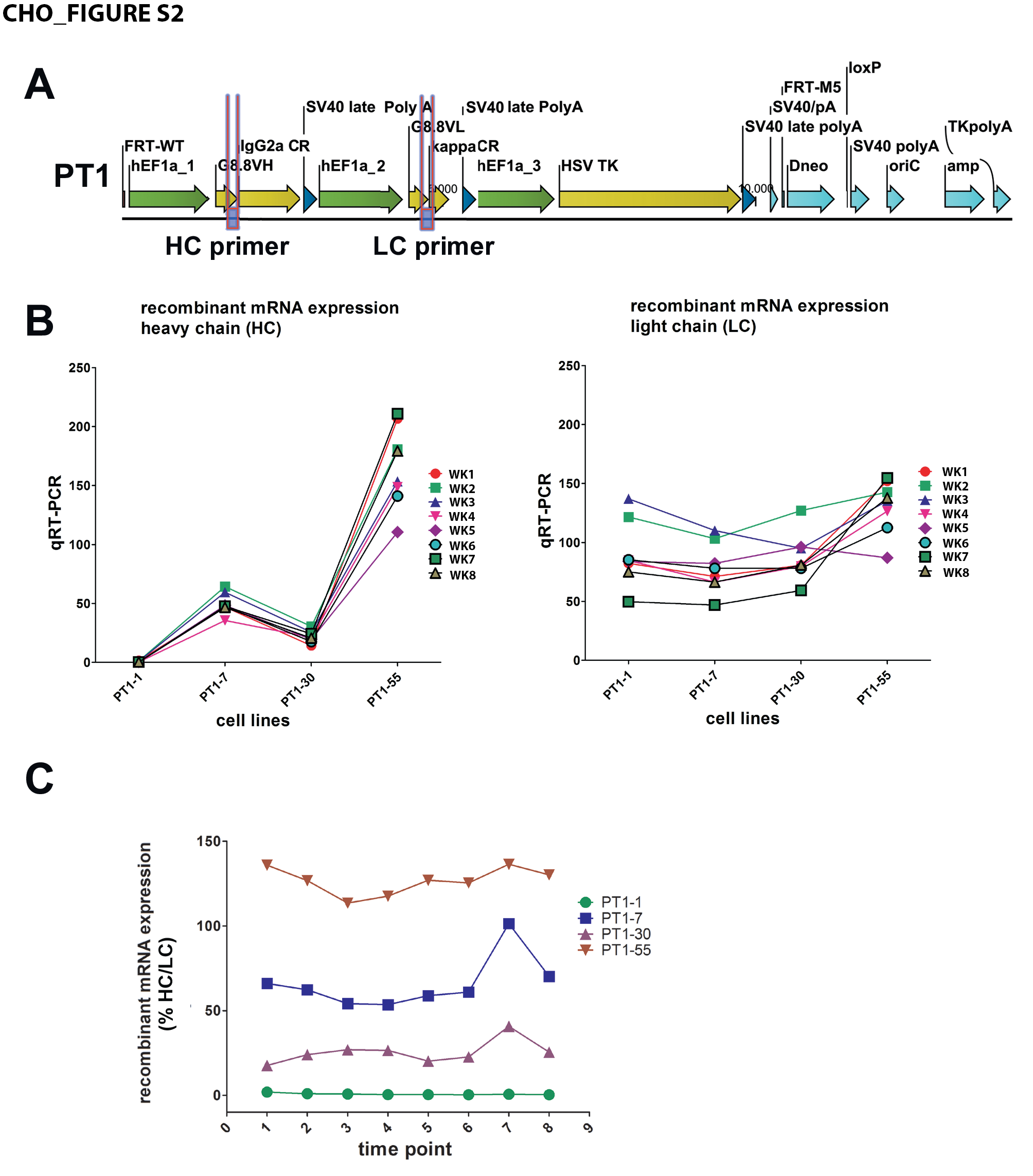

Supplement: Additional file 2: Figure S2. — Recombinant mRNA expression in four different PT1-CHO cell lines. (A) A schematic diagram of the PT1 vector showing the location of fragments amplified by the qRT-PCR heavy chain (HC) and the light chain (LC) primers. (B) mRNA expression as measured by the HC (left panel) and by LC (right panel) primers after a two-month continuous adherent culturing and passaging (P56 to P72) in 10 % FCS. (C) mRNA expression of same samples based on percentage of the HC qRT-PCR values (B, left panel) over that of LC values (B, right panel). (TIF 753 kb) [file 12896_2016_238_MOESM2_ESM.tif]

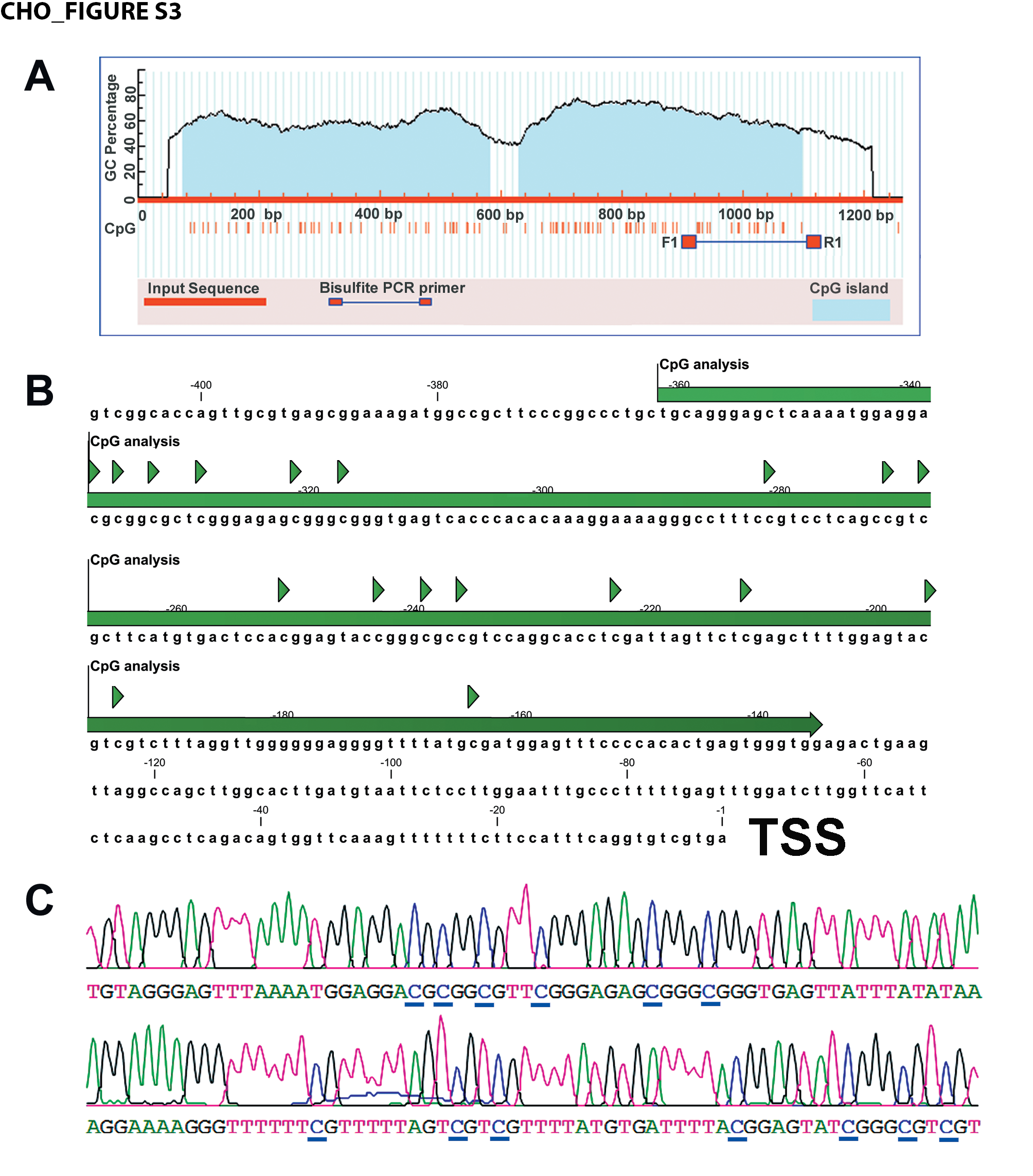

Supplement: Additional file 3: Figure S3. — DNA methylation analysis in the EEF1A1 promoter region in PT1-CHO cell lines. (A) A schematic representation of the two CpG islands and bisulfite sequencing primers as identified using MethPrimer (http://www.urogene.org/methprimer/index1.html) (B) Sequence of the analyzed fragment (231-bp) and the analyzed CpGs (arrows). The last nucleotide in the 1261-bp EEF1A1 promoter towards the transcription start site (TSS) was designated as −1. (C) Partial Sanger sequence electropherogram with methylated CpGs underlined. (TIF 2035 kb) [file 12896_2016_238_MOESM3_ESM.tif]

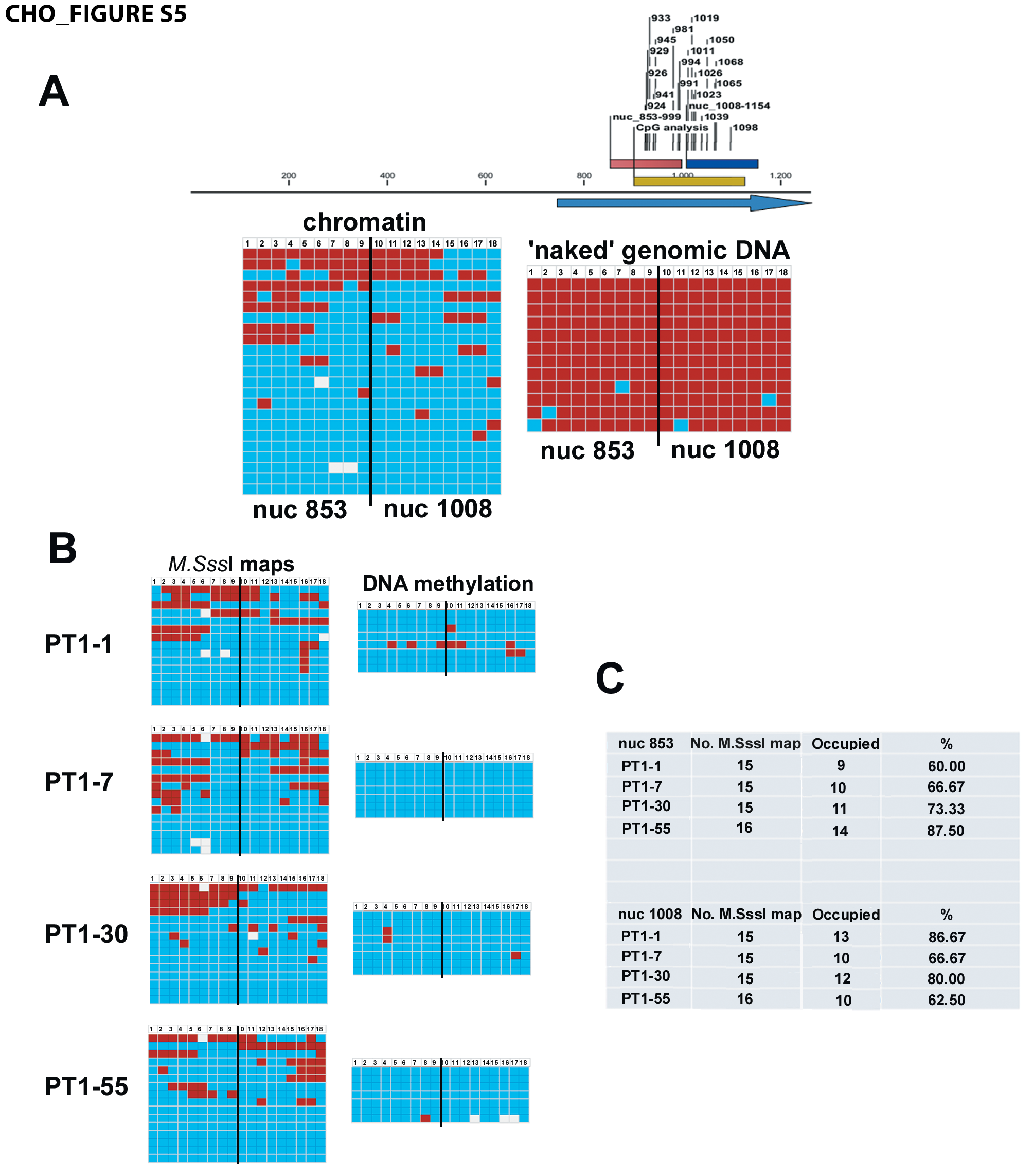

Supplement: Additional file 4: Figure S5. — M.SssI chromatin mapping on the EEF1A1 promoter in PT1-CHO cell lines. (A) Schematic annotation of the promoter region and the comparison of results in chromatin and ‘naked’ genomic DNA after M.SssI treatment. In ‘naked’ genomic DNA, most CpGs are methylated, while the unmethylated ones are random. In the chromatin, stretches of unmethylated or protected CpGs are evident to suggest occupancy of a nucleosome. (B) M.SssI chromatin maps of the four PT1-CHO cell lines (left panel); corresponding DNA methylation pattern (bisulfite-treated genomic DNA only), indicating endogenous methylation on the same cell lines (right panel). Methylated CpGs, red); Unmethylated CpGs, blue). (C) Nucleosome occupancy in the two analyzed nucleosomes Nuc 853 (nt 853–999) and Nuc 1008 (nt 1008–1154). (TIF 1777 kb) [file 12896_2016_238_MOESM4_ESM.tif]

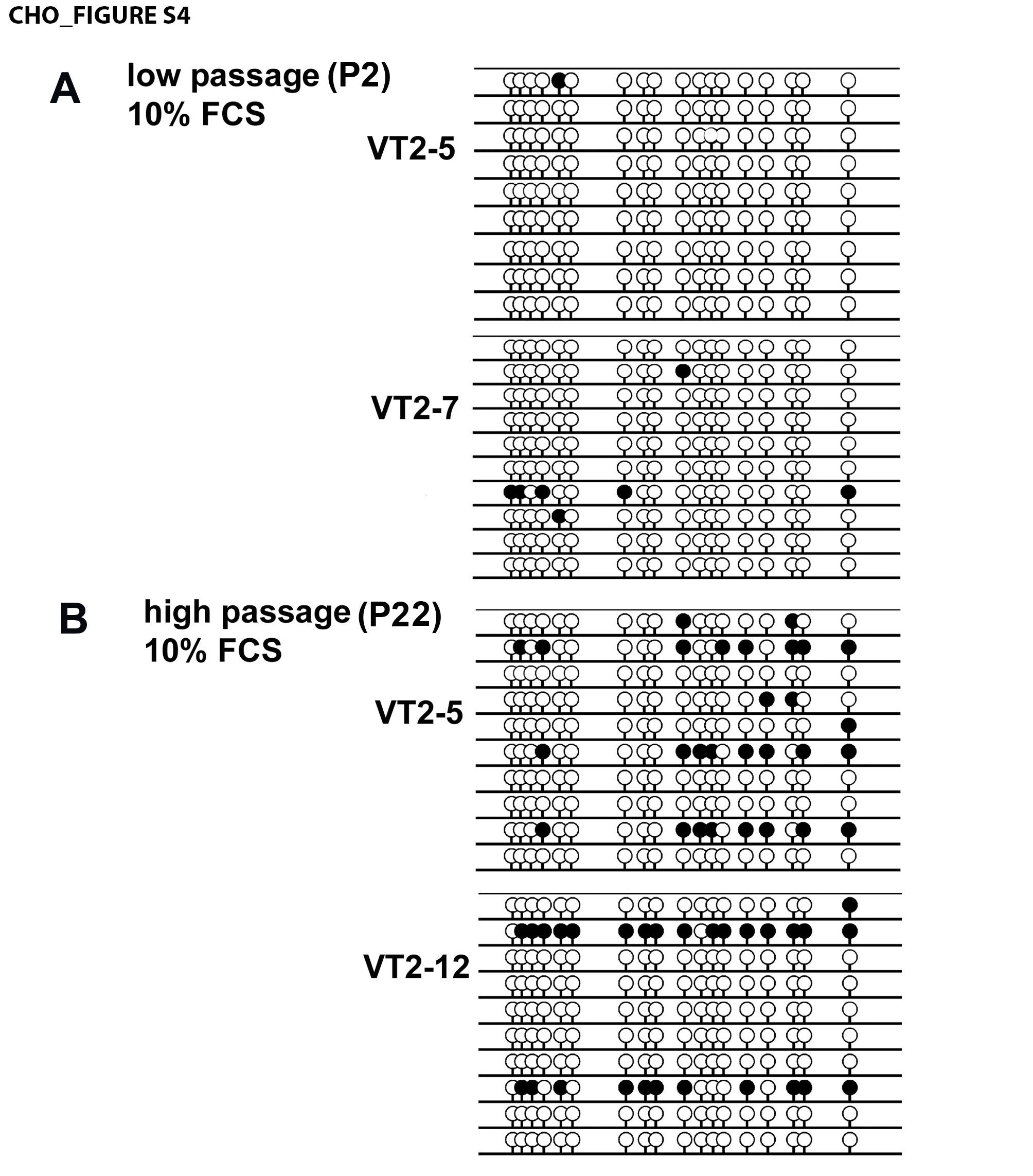

Supplement: Additional file 5: Figure S4. — DNA methylation analysis in the EEF1A1 promoter region in VT2-CHO cell lines, at low vs. high passage. Methylated CpGs (filled lollipops), unmethylated CpGs (unfilled lollipops). (TIF 738 kb) [file 12896_2016_238_MOESM5_ESM.tif]

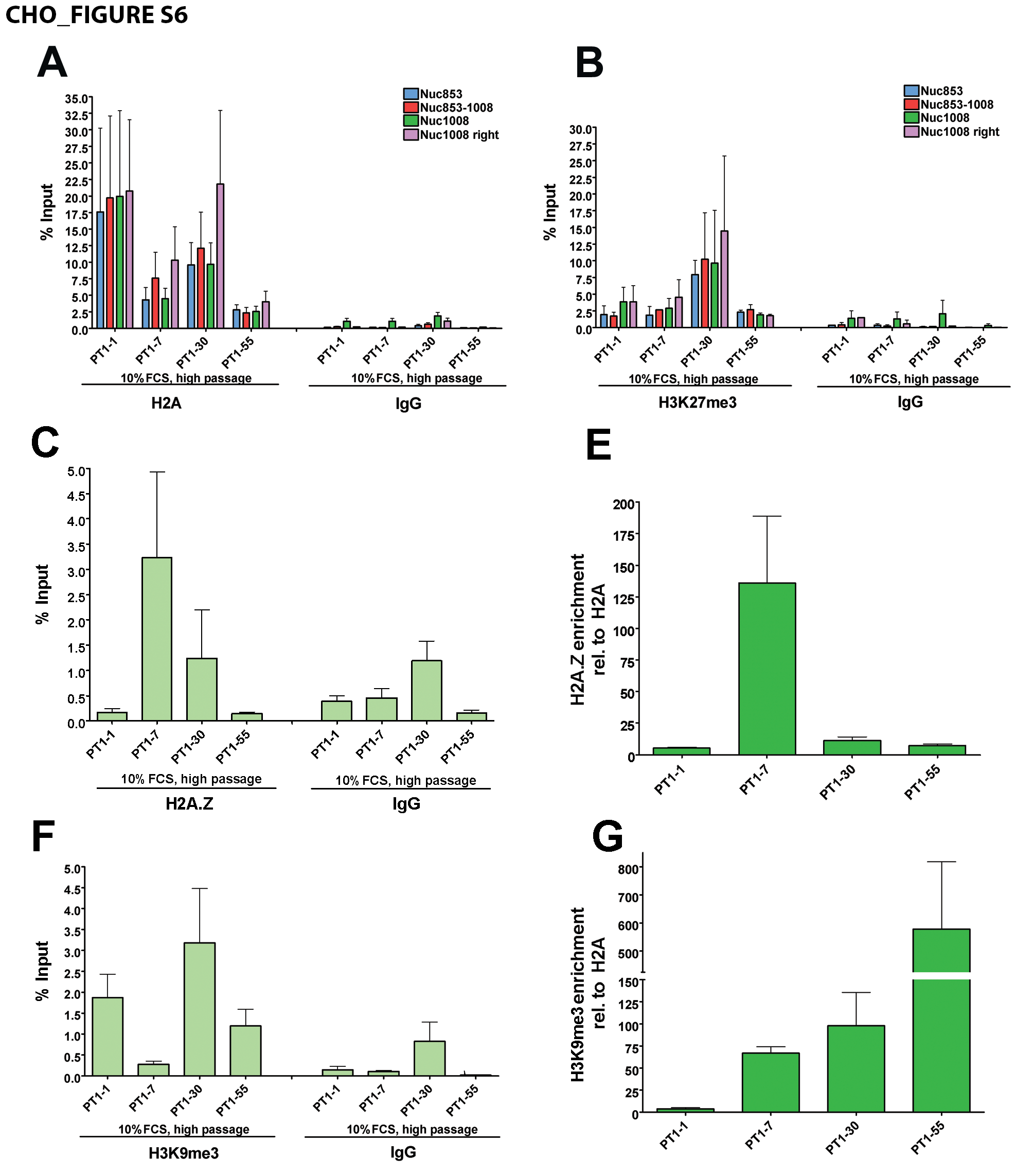

Supplement: Additional file 6: Figure S6. — ChIP enrichments in PT1-CHO cell lines. (A) H2A enrichments in the different PT1-CHO cell lines and four qPCR primer pairs. The data were analyzed by two-tailed ANOVA, which found statistically significant differences in H2A enrichment (H2A nucleosome occupancy) among the cell lines (** P = 0.0070), but not differences resulting with the qPCR primers (nucleosome). Results are presented as % input calculated from Ct values, and the means and SEM of n = 12 independent experiments involving four primer pairs along two predicted nucleosome positions in the EEF1A1 promoter region. Also shown are the qPCR values obtained in the four primer pairs for IgG, which served as control for the ChIP experiment. (B) ChIP with H3K27me3 and control IgG obtained after qPCR with four primer pairs. (C, E, F, G) ChIP with H2A.Z, H3K9me3, and control IgG obtained by % input DNA, left panels), and H2A.Z and H3K9me3 after further normalization to H2A (right panels). Data represent means and SEM of n = 3 independent experiments. (TIF 799 kb) [file 12896_2016_238_MOESM6_ESM.tif]
